# Abilities, Motivations, and Opportunities of Furloughed Employees in the Context of Covid-19: Preliminary Evidence From the UK

**DOI:** 10.3389/fpsyg.2021.635144

**Published:** 2021-03-11

**Authors:** Joanna Maria Szulc, Rachael Smith

**Affiliations:** Huddersfield Business School, University of Huddersfield, Huddersfield, United Kingdom

**Keywords:** COVID-19, furlough, ability, motivation, opportunity, cutback management, HR management

## Abstract

The Covid-19 global pandemic is a crisis like no other, forcing governments to implement prolonged national lockdowns in an effort to limit the spread of the disease. As organizations aim to adapt and remain operational, employers can suspend or reduce work activity for events related to Covid-19 and claim government support to subsidize employee wages. In this way, some employees are placed on furlough (i.e., temporary unemployment) as opposed to being made redundant. While the impact of such schemes on global economy attracted much attention, their micro-level impact on individual employees is still unknown. Building on the *ability-motivation-opportunity* (AMO) framework, this pilot study explores how employees' perceptions of abilities, motivation, and opportunities are affected as a result of furlough. Rapid ethnography including interviews, observations, and document analysis in a British organization provided insights into the perceptions and experiences of employees put on furlough and highlighted that all three elements of AMO are affected by the current situation, either positively or negatively. We identify theoretical contributions and suggest a number of AMO enhancing practices in the context of furlough.

## Introduction

Furloughs have been used by governments as a job saving tool since the 2012 economic recession (Lee and Sanders, 2013). They allow employers to place individuals on temporary leave and act as a cost-saving exercise. As a relatively new concept in the United Kingdom (UK), furlough was suggested by the government to assist the economy by limiting the number of redundancies that occurred as a result of a Covid-19 pandemic. Organizations that were unable to maintain their workforce were encouraged to furlough employees rather than make redundancies and apply for the Coronavirus Job Retention Scheme, which reimbursed employers with 80% of workers' wages.

Furlough may influence many aspects of life on a personal and organizational level, as employees aim to retain employment whilst adapting to changes to working conditions such as reduced pay and hours. Although research exists to examine how individuals deal with the job insecurity resulting from national crises (Adkins et al., 2001) and how such crisis management affects organizational and management levels (James et al., 2011; Stoker et al., 2019), our knowledge of its effect on individual employees is scant (Perrewé et al., 2012). While this is inevitably linked to the recency of this unprecedented event and its unique circumstances (Shoss et al., 2021), greater understanding of the impact of furlough on employees would enable organizations to develop practices and processes encouraging development and increasing motivation and commitment in such difficult times.

The aim of this research is therefore to explore the impact of furlough on employee's perceptions of ability, motivation, and opportunity through the lens of the AMO theoretical framework (Boxall and Purcell, 2003). According to the theory, individual functioning is largely dependent upon one's ability, motivation, and opportunity to participate in an organizational life. By exploring how these three aspects are affected by a decision to be sent on furlough, our study contributes to the existing literature in a number of ways.

First, the reported study enhances our knowledge of employee behaviors during times of crisis. As organizations over the decades have adapted in times of crises including the Asian Financial Crisis or the Global Financial Crisis, it is the Covid-19 crisis, in particular, that had a revolutionary effect on business-as-usual. However, the evidence on its impact is only in its infancy (El Keshky et al., 2020; Teng et al., 2020). Second, we adopt a well-established AMO framework in a novel context of furloughed employees. In doing so we enhance existing understanding of the abilities, motivations, and opportunities of furloughed employees and their potential impact on organizationally-relevant outcomes. Third, this paper explains how perceptions of, and reactions to the same Human Resources (HR) practices may differ for furloughed employees. Hence, it addresses the call by Cafferkey et al. (2019) to move away from universal HR as a route to positive employee outcomes. By exploring the impact of furlough on the work experiences of affected individuals, we facilitate a more accurate reflection of organizational reality (see also Kinnie et al., 2005) particularly in an unstable and dangerous business environment (Shehzad et al., 2020).

## Literature Review

In light of the significant gap in the existing literature regarding the effects of furlough in the times of Covid-19, we can build on some of the existing research investigating employee behaviors during times of crisis (e.g., Adkins et al., 2001; Stoker et al., 2019) or cutbacks (Robinson and Rousseau, 1994). For instance, Lee and Sanders (2013) argued that cutback policies challenge employees' perceptions of positive self-worth by reducing confidence in own abilities and that they decrease opportunities for self-advancement. Additionally, it was found that such reductions in organizational spending will often lead to significant decreases in employee morale (Cameron, 1994; Tselikis, 2001) and trust in their organization (Feldheim, 2007).

Despite furloughs being a cost-saving exercise that can save jobs in the long-run, they are regularly perceived as a unique form of job insecurity (Halbesleben et al., 2013). The threat of a loss of resources is often associated with higher levels of emotional exhaustion experienced by employees (Bellairs et al., 2014) which could be further strengthened when individuals are isolated from social and emotional interactions at work (Bentein et al., 2017). Such severe affective experiences negatively impact motivation and job satisfaction (Carlson et al., 2011).

Alternatively, Halbesleben et al. (2013) explains how furloughed employees may be motivated to seek resources that mitigate their experienced losses. They suggest that certain recovery experiences may mitigate the overly negative impact of furlough. However, we still do not know what exactly the impact of furlough following the Covid-19 outbreak is on individuals beyond the figures demonstrating that furloughed employees are three times more concerned about the likelihood of losing their jobs than those unaffected by furlough (Chartered Institute of Personnel Development, 2020a). Consistently, more research is needed to explore the effects of furlough on individuals to mitigate the potential damage it may bring.

Our approach to examining the impact of furlough on individual employees is based on the AMO model which implies that HR practices should improve our abilities, boost motivation, and provide opportunities to meet organizational objectives (Jiang et al., 2012). In fact, the specific HR practices focused on the abilities, motivation and opportunities have been reported as an organizational tool to best endure furloughs in the past (Bellairs et al., 2014; Baranik et al., 2019). We do not know, however, how the specific components within a model have been impacted in the wake of the current global pandemic.

## Method

To provide a reasonable understanding of furloughed employees and their perceptions on their abilities, motivations, and opportunities and to understand how these matter to the design of the company's HR strategies, a qualitative rapid ethnography methodology was employed (Tate, 2020). The core elements included constraining the research focus and scope to one organization were furlough was introduced and using key informants. Since the logic of theoretical sampling lies in the selection of information-rich cases studied in depth (Morse and Clark, 2019), the choice of an organization currently undergoing furloughs enabled the authors to impute the theoretical aspects of the research. The second author was an HR manager in a leading air technology organization in Northern England tasked with the planning and coordination of furlough.

The methods within a rapid ethnography included onsite observations, document analysis, and 15 in-depth semi-structured interviews with furloughed employees. All participants sent on furlough were purposefully included in the sample with the exception of the four participants whose furlough was shorter than 3 weeks and who were consequently made redundant. Since the whole population of employed furloughed individuals was included in the sample, our research design facilitated the provision of comprehensive perspectives. Of the respondents, 11 were male and four were female. The average tenure was 4.5 years (shortest 1 year; longest 18.5 years). All furloughs were mandatory and resulted in 20% reduction in salary. The average length of furlough was 5.5 months (shortest 2 months; longest 9 months).

The data collection process started in March 2020 when furlough was introduced and was complete in November 2020. The project gained ethical approval in March when the observations commenced. These observations were performed as part of the second author's everyday work. Most interviews took place between August and September. The second author contacted individual employees sent on furlough directly by email with the information about the project and an interview invitation. Potential participants were assured of confidentiality and a voluntary nature of the study. All 15 contacted individuals expressed interest in taking part. Interviews were conducted online via Teams software and lasted on average 30 min (shortest 20 min; longest 50 min). Interview questions were designed to find information on individuals' experiences of furlough with special emphasis being given to its perceived impact on their abilities, motivation, and opportunities. Each interview was recorded and transcribed verbatim. The process resulted in 225 pages of transcripts accompanied by the second author's reflexive notes.

Aligned with the rapid ethnography approach, the informal analysis commenced during the observations. Observation field notes were critically reflected on by both authors as part of an iterative process. The formal analysis of the interview material was conducted collaboratively by the two authors using Template Analysis (King, 2012). Transcripts were first inspected to account for possible mistakes by both authors. Themes were developed independently and then cross-checked ensuring inter-coder-reliability (O'Connor and Joffe, 2020). While the second author was assigned primary responsibility for creating, updating, and revising the codes (Guest and MacQueen, 2008), both authors engaged in intensive discussions to reach a consensus on the final coding template during documented team meetings to increase the reliability of the study (Saldaña, 2021). Since the second author had a pre-understanding and own assumptions about the research context, she maintained a reflexive diary which is generally associated with higher quality research (Maxwell, 2012). In fact, the insider's position and prolonged engagement with the case study organization, increased the sense of an overall embeddedness and awareness of the studied context and thus increased the validity of the study (Morse et al., 2002). To minimize the potential negative impact of the first author's position and further improve the validity of the study, we engaged in peer debriefing (Maxwell, 2013) and conducted member checks with a fifth of respondents (Caretta, 2016).

## Results

### Effect of Furlough on Employee Perceptions of Abilities

One's skillset was perceived as a determining factor when selecting employees to be furloughed. For instance, Participant I suggested “*X worked a lot quicker than me and knew more than me when it came down to customer queries so they chose her to stay in work*.” It was commonly reported that colleagues who were not sent on furlough had more skills and abilities (“*I knew the admin side but he also knew how to build the KITS in the warehouse*,” Employee J) and were therefore more “*needed*” (Employee F).

A further theme that emerged within the study suggested that personal circumstances such as health or childcare provisions can affect the way in which one's abilities to complete work are perceived by employees themselves:

*“Because I had nowhere for the kids as the schools closed I was stressed about how I was going to manage juggling it all, especially if I would have been working from home”*—Employee M

Lack of childcare as a result of Covid-19 lockdown restrictions, affected employee M's ability to carry out her day to day role. This, in fact, was a common theme in the UK, where 67% of keyworkers were forced to reduce working hours due to lack of childcare (Office for National Statistics, 2020). Indeed, working mothers were described as the “sacrificial lambs of the coronavirus pandemic” (Topping, 2020). Struggling with the increased demands of working from home alongside childcare may negatively impact on one's abilities to complete the job in contrast to those without caring responsibilities and thus further enhances the gap between those two groups (Kelly and Senior, 2020). Indeed, 52% of working mothers surveyed in July 2020 perceived they were treated negatively by their employer, due to lack of childcare during Covid-19 (Howlett, 2020).

Another theme that emerged along with the interview progress was participants undertaking proactive forms of learning and development whilst furloughed. For instance, Employee A said he “*read up on a few manuals to kill time and make sure I was up-to-date when it was time to come back*” and Employee O suggested that “*our industry is always changing so it was a chance to make sure I was up to date*.”

Interestingly, some participants admitted that they self-sought training and development whilst furloughed to enhance their skillset should they need to seek alternative employment (“*it is something extra on my CV should things get worse here and we have job cuts*,” Employee A). Employee N, for instance, diversified his skillset by completing training in alternative field as a response to upcoming potential lockdowns or redundancies:

*“I have done CAD training already but if we go into a full lockdown again it's something you can do from home, or if I needed to change career completely I have it as a back-up”*—Employee N

Existing literature promotes the benefits of learning and development opportunities for both employers and employees (Chartered Institute of Personnel Development, 2020b) and it was apparent that participants within the study recognized the importance of obtaining new qualifications in an attempt to increase their human capital within their organization but also to increase employability externally.

### Effect of Furlough on Employee Perceptions of Motivation

The effect of furlough on individuals' perceived motivation was reported to be mixed. On the one hand, several participants suggested that their perceptions of job security had been negatively affected due to furlough. For instance, in comparison to non-furloughed employees, research participants “*felt replaceable”* (Employee L) and indicated that the risk of redundancy had increased for them as a result of being furloughed:

*“I did think that if I stayed off too long you might have coped without me so if it came down to it, I would be the one to go (redundancy) rather than one of the other lads”*—Employee A

In addition to the above comment, participants regularly highlighted that the risk of redundancy was increased as furloughed employees would not have the required knowledge or opportunities that employees remaining within the workplace had gained during this time. This suggests that furlough may negatively impact *abilities, opportunities, and motivation* simultaneously. This is in line with the existing literature pointing out to the interactive and overlapping nature of the three elements of AMO (Kellner et al., 2016).

Increased feelings of job insecurity resulting from being furloughed link back to the existing literature which suggests that job insecurity is a stressor which negatively impacts behaviors and motivation of employees displayed through decreased job satisfaction and increased intention to leave the organization (Blyton and Bacon, 2001). Consistently, the sense of powerlessness and uncertainty as a result of Covid-19 pandemic and furlough could have enhanced the threat of job insecurity with these furloughed participants.

The salary reduction associated with furlough was a further theme that emerged in the data analysis. While individuals who were furloughed incurred a 20% loss of earnings, only participants considered to be in lower-paid roles commented how severely they were impacted by the salary reduction. For instance, Employee C commented:

*“I did start to worry about how long I could manage (financially) or when I was going to be coming back in, I think one of the hardest parts is when you don't know if you will be back in this month or not”*—Employee C

Existing research suggests that salary reductions will be particularly demotivating for individuals in lower-paid roles (Kahneman and Deaton, 2010). In line with this line of inquiry, we found that for those in higher-skilled and better paid roles, reduction in pay did not appear to affect them as much as they anticipated. In fact, participants with health problems and on statutory sick leave, ended up with higher payments when furloughed. This was reported to make them feel “*extremely relieved”* (Employee A) and pointed out to the positive effect of furlough.

A potential lack of a negative impact on participants' perceived motivation may be further related to all interviewees being clear on the reasons for furloughing staff and perceiving this more positively than job cuts: “*A lot of places were just laying off. So I was glad that furlough was even an option”* (Employee F). Employee G provided a more detailed explanation where he suggested that being sent on furlough was presented by an organization as an opportunity to contribute to organizational cost-saving activities and to protect the roles of others:

*“I took it that it was a good thing as when I was told about me being furloughed it was like I was doing a good thing for the company, helping out (…) I was helping because I was in a role where I could be furloughed so they could make those cost savings, I was protecting people in roles that couldn't (be furloughed)”*—Employee G

Participants who did not raise concerns regarding job security due to furlough further revealed that they were communicated the financial position of the organization either during their initial consultation or whilst furloughed:

*“When I was told that we had won this contract, it made me less worried as financially that was good for us, for the organization. There was also a note from a managing director appreciating everyone's contributions to winning this project, including the furloughed staff”*—*Employee K*

It appears that framing the decision to send a specific individual on furlough by emphasizing their important role in what is seen as a cost-saving exercise and clearly communicating the financial position of the business could have reduced the perceived threat of redundancies and thus positively impacted the participants' views of job security and their motivation.

### Effect of Furlough on Employee Perceptions of Opportunity

Participants reported that while on furlough they had opportunities to voice their views through informal discussions with colleagues and approaching line managers. They all confirmed they remained in contact with at least one other employee. Most of the participants stated they received at least two telephone updates from their line manager with updates on the likelihood of furlough continuing. Employee K commented:

*“I knew what was going on from my team. I then got updates about potential return dates from my manager. Also, because I received the newsletter, I felt more included as I knew what was going on”*—Employee K

Adequate opportunities to participate in organizational life and take an active role raising concerns or providing suggestions were further found to have a positive impact on participants' commitment to the organization during times of uncertainty. While four participants acknowledged they had searched for employment while on furlough to evaluate the current labor market and to assess what type of industry vacancies were available, the majority of participants did not look at alternative employment options despite insecure job environment. It is these employees who emphasized how satisfied they were with the level of contact they received whilst furloughed.

Participants' commitment to the organization during times of uncertainty could be explained through the lens of the social exchange theory (Cropanzano et al., 2017). It suggests that providing employees with the opportunities to participate in organizational life through increased communication results in increased organizational commitment due to perceptions of favorable treatment (Morales-Sánchez and Pasamar, 2019). This appears particularly relevant in the times when “the routine” (Employee L) and “people side and interaction” (Employee K) were generally reported to be lost as a result of furlough. As succinctly illustrated below, the most direct effect on furlough appears to be the lost opportunity to engage in every-day work and the social element of this:

*“I miss working, getting on sites, and the fun bits like seeing people at work. I can't come into the office so I'm losing social time too”*—Employee G

## Discussion

The aim of this study was to explore the impact of furlough on employee's perceptions of their abilities, motivations, and opportunities. In line with the existing literature that presents furloughs as a job saving action (Lee and Sanders, 2013) superior to other cutback options (Green, 2010), this study revealed furlough was relatively positively received as a cost-saving exercise. It was reported that furlough opened up new opportunities for proactive up-skilling and exploring new employment opportunities. This is in contrast to most recent statistics suggesting that, in the times of pandemics, nearly a third of UK workers lack confidence in their abilities and how these can be transferred to different sectors (Donnelly, 2021). Importantly, furlough was still reported by our research participants as a threat to job security and concerns of being at an increased risk of redundancy as a direct result of being furloughed were raised. This finding aligns with the existing research suggesting that the threat of furlough and associated risks of losing one's job creates demands of employees that can result in increased feelings of strain (Halbesleben et al., 2013; El Keshky et al., 2020). We also found that absence from workplace as a result of being furloughed may lead to perceptions of missing out on opportunities to develop oneself and one's abilities. This finding should be considered in the light of research suggesting that individuals can mitigate the experienced resource loss through recovery experiences (Sonnentag, 2001). Finally, we found that potentially negative effects of furlough on employees' perceptions of ability, motivation and opportunity were at least partly alleviated when employers maintained regular communication with those on furlough and assured them of the value they bring to the organization. This is aligned with the existing research suggesting that when employees view the process of furlough as egalitarian and appropriate, they feel fortunate to preserve their positions and remain loyal to the organization (see Lee and Sanders, 2013).

Theoretically, the reported research contributed toward the existing lacuna of knowledge on the factors that influence employee perceptions of their abilities, motivations, and opportunities as a result of furlough and in the times of uncertainty (Davies, 2020). More specifically, existing limited research in this area focused on unpaid mandatory furlough of varied duration and more *ad-hoc* nature to combat the effects of global financial crisis (Halbesleben et al., 2013; Lee and Sanders, 2013). We extend this line of research to include furlough and its effects in a novel context. Secondly, since furloughs are considered as time away from work (Halbesleben et al., 2013), our research further extends the existing line of inquiry that suggests such time alleviates workplace stressors and enhances employee well-being (Sonnentag, 2005). Contrary to these common assumptions, our findings suggest that furlough is an exceptional time away from work–while it brings opportunities to develop oneself, it is also perceived more negatively as a threat to one's job security.

Practically, our findings suggest that an effective consultation clarifying the rationale for furlough and why employees had been selected will positively impact perceptions of fairness. Additionally, clarifying the financial position and length of expected furlough is likely to alleviate the potentially negative impact of furlough on one's motivation through the decreased feelings of uncertainty. We further recommend flexible furlough utilization, i.e., encouraging employers to rotate employees on furlough. This flexibility not only provides benefits to mental health and decreases the time spent away from the organization, but it also alleviates concerns regarding the negative impact on employee opportunities. As the impact of reduction in pay was found to negatively impact low paid workers disproportionally to their colleagues, organizations can discuss furlough with employees and, where possible, request furlough volunteers who would perceive the decision positively. Circumstances which are likely to negatively impact employees such as lack of childcare or those who would otherwise be required to self-isolate should also be considered. Finally, we found that furloughed individuals often considered their skills to be inferior to the skills of their non-furloughed colleagues. This may lead to low self-esteem and confidence problems (Shack et al., 2018) which, if not addressed, may influence overall organizational climate by diving the workforce and negatively affecting their relationships (Szulc, 2020). Consistently, employers may consider implementing return-to-work interviews where employee's value and contribution to the organization is emphasized.

## Limitations and Future Research Directions

Our research was limited to a small sample and the UK context and does not allow for generalizations. However, qualitative approach provided us with some in-depth contextual information that moved beyond a simple set of variables and emphasized the importance of context when proposing explanations. This research can therefore act as a pilot, preliminary study to future research testing the proposed findings in larger scale samples, across industries and geographic countries. Additionally, since a second author is an HR professional in the studied company, issues of neutrality in the data collection process may arise. To minimize the risk, we engaged in a number of strategies including maintaining a reflexive diary, peer debriefing, and member checks (described in detail in a Method section). We hope that such strategies, in fact, increased the sense of an overall embeddedness and awareness of the studied context and eventually improved the validity of the study. Further research activity in this area should considerably contribute toward preventing long-lasting emotional trauma and disturbance to the psychological well-being of employees and toward creating sustainable development for well-being in organizations (see Safraz et al., 2020).

## Data Availability Statement

The raw data supporting the conclusions of this article will be made available by the authors, without undue reservation.

## Ethics Statement

The studies involving human participants were reviewed and approved by University of Huddersfield, Business School Research Ethics Committee (BSREC). The patients/participants provided their written informed consent to participate in this study.

## Author Contributions

JS: introduction, analysis, methodology, discussion, revising work, and proofreading. RS: data collection, analysis, and results. All authors contributed to the article and approved the submitted version.

## Conflict of Interest

The authors declare that the research was conducted in the absence of any commercial or financial relationships that could be construed as a potential conflict of interest.

## References

[B1] Adkins C. L. Werbel J. D. Farh J. L. (2001). A field study of job insecurity during a financial crisis. Group Organ. Manag. 26, 463–483. 10.1177/1059601101264004

[B2] Baranik L. E. Cheung J. H. Sinclair R. R. Lance C. E. (2019). What happens when employees are furloughed? a resource loss perspective. J. Career Dev. 46, 381–394. 10.1177/0894845318763880

[B3] Bellairs T. Halbesleben J. R. B. Leon M. R. (eds.). (2014). A multilevel model of strategic human resource implications of employee furloughs, in Research in Personnel and Human Resources Management (Bingley: Emerald Group), 99–146.

[B4] Bentein K. Garcia A. Guerrero S. Herrbach O. (2017). How does social isolation in a context of dirty work increase emotional exhaustion and inhibit work engagement? a process model. Pers. Rev. 46, 1620–1634. 10.1108/PR-09-2016-0227

[B5] Blyton P. Bacon N. (2001). Job insecurity: a review of measurement, consequences, and implications. Hum. Relat. 54, 1223–1233. 10.1177/0018726701549004

[B6] Boxall P. F. Purcell J. (2003). Strategy and Human Resource Management. Basingstoke: Palgrave Macmillan.

[B7] Cafferkey K. Heffernan M. Harney B. Dundon T. Townsend K. (2019). Perceptions of HRM system strength and affective commitment: the role of human relations and internal process climate. Int. J. Hum. Resour. Manag. 30, 3026–3048. 10.1080/09585192.2018.1448295

[B8] Cameron K. S. (1994). Strategies for successful organizational downsizing. Hum. Resour. Manag. 33, 189–211. 10.1002/hrm.3930330204

[B9] Caretta M. A. (2016). Member checking: a feminist participatory analysis of the use of preliminary results pamphlets in cross-cultural, cross-language research. Qual. Res. 16, 305–318. 10.1177/1468794115606495

[B10] Carlson D. Kacmar K. M. Zivnuska S. Ferguson M. Whitten D. (2011). Work-family enrichment and job performance: a constructive replication of affective events theory. J. Occup. Health Psychol. 16, 297–312. 10.1037/a0022880 21728437

[B11] Chartered Institute of Personnel Development (2020a). CIPD Good Work Index 2020: UK Working Lives Survey. Available online at: https://www.cipd.co.uk/Images/good-work-index-full-report-2020-2_tcm18-79210.pdf (accessed February 05, 2021).

[B12] Chartered Institute of Personnel Development (2020b). Learning and Development. Available online at: https://peopleprofession.cipd.org/profession-map/specialist-knowledge/learning-development (accessed November 10, 2020).

[B13] Cropanzano R. Anthony E. L. Daniels S. R. Hall A. (2017). Social exchange theory: a critical review with theoretical remedies. Acad. Manag. Ann. 11, 1–38. 10.5465/annals.2015.0099

[B14] Davies J. (2020). Implications for HRD practice and impact in the COVID-19 era. Hum. Resour. Dev. Rev. 20, 3–8. 10.1177/1534484320977426

[B15] Donnelly K. (2021). Building Bridges Towards Future Jobs. Available online at: https://www.cityandguildsgroup.com/-/media/cgg-website/documents/building-bridges-towards-future-jobs-report-pdf.ashx?la=enandhash=07F6AE148AF11FACE03085A57B6F4243407C18E8 (accessed February 05, 2021).

[B16] El Keshky M. Basyoun S. S. Al Sabban A. M. (2020). Getting through COVID-19: the pandemic's impact on the psychology of sustainability, quality of life, and the global economy—a systematic review. Front. Psychol. 11:585897. 10.3389/fpsyg.2020.585897 33281683 PMC7688781

[B17] Feldheim M. A. (2007). Public sector downsizing and employee trust. Int. J. Public Admin. 30, 249–270. 10.1080/01900690601117739

[B18] Green M. Z. (2010). Unpaid furloughs and four-day work weeks: employer sympathy or a call for collective employee action? Conn. Law Rev. 42, 1–35. Available online at: https://scholarship.law.tamu.edu/cgi/viewcontent.cgi?article=1053&amp;context=facschola

[B19] Guest G. MacQueen K. M. (2008). Handbook for Team-Based Qualitative Research. New York, NY: AltaMira Press.

[B20] Halbesleben J. R. B. Wheeler A. R. Paustian-Underdahl S. C. (2013). The impact of furloughs on emotional exhaustion, self-rated performance, and recovery experiences. J. Appl. Psychol. 98, 492–503. 10.1037/a0032242 23506412

[B21] Howlett E. (2020). Half of Mums Made Redundant During Covid-19 Cite Lack of Childcare. People Management. Available online at: https://www.peoplemanagement.co.uk/news/articles/half-of-mums-made-redundant-during-covid-19-cite-lack-of-childcare (accessed November 10, 2020).

[B22] James E. H. Wooten L. P. Dushek K. (2011). Crisis management: informing a new leadership research agenda. Acad. Manag. Ann. 5, 455–493. 10.5465/19416520.2011.589594

[B23] Jiang K. Lepak D. P. Hu J. Baer J. C. (2012). How does human resource management influence organisational outcomes? a meta-analytic investigation of mediating mechanisms. Acad. Manag. J. 55, 1264–1294. 10.5465/amj.2011.0088

[B24] Kahneman D. Deaton A. (2010). High income improves evaluation of life but not emotional well-being. Proc. Natl. Acad. Sci. U.S.A. 107, 16489–16493. 10.1073/pnas.1011492107 20823223 PMC2944762

[B25] Kellner A. Townsend K. Wilkinson A. Lawrence S. A. Greenfield D. (2016). Learning to manage development experiences of hospital frontline managers. Hum. Resour. Manag. J. 26, 505–522. 10.1111/1748-8583.12119

[B26] Kelly S. Senior A. (2020). Towards a feminist parental ethics. Gend. Work Organ. 1. 10.1111/gwao.12566. [Epub ahead of print].

[B27] King N. (2012). Doing template analysis, in Qualitative Organizational Research, eds Symon G. Cassell C. (London: Sage), 426–450.

[B28] Kinnie N. Hutchinson S. Purcell J. Rayton B. Swart J. (2005). Satisfaction with HR practices and commitment to the organisation: why one size does not fit all. Hum. Resour. Manag. J. 15, 9–29. 10.1111/j.1748-8583.2005.tb00293.x

[B29] Lee S. Sanders R. M. (2013). Fridays are furlough days: the impact of furlough policy and strategies for human resource management during a severe economic recession. Rev. Public Pers. Admin. 33, 299–311. 10.1177/0734371X13477426

[B30] Maxwell J. (2012). A Realist Approach to Qualitative Research. Thousand Oaks, CA: Sage.

[B31] Maxwell J. (2013). Qualitative Research Design: An Interactive Approach, 3th Edn. Los Angeles, CA: Sage.

[B32] Morales-Sánchez R. Pasamar S. (2019). How to improve organisational citizenship behavior by combining ability, motivation, and opportunity: the moderator role of perceived organisational support. Employee Relat. 42, 398–416. 10.1108/ER-04-2019-0169

[B33] Morse J. Barrett M. Mayan M. Olson K. Spiers J. (2002). Verification strategies for establishing reliability and validity in qualitative research. Int. J. Qual. Methods 1, 13–22. 10.1177/16094069020010020218626033

[B34] Morse J. Clark L. (2019). The nuances of grounded theory sampling and the pivotal role of theoretical sampling, in The SAGE Handbook of Current Developments in Grounded Theory, eds Bryant A. Chamraz K. (London: Sage), 145–166.

[B35] O'Connor C. Joffe H. (2020). Intercoder reliability in qualitative research: debates and practical guidelines. Int. J. Qual. Methods 19, 10.1177/1609406919899220

[B36] Office for National Statistics (2020). Parenting in Lockdown: Coronavirus and the Effects on Work-Life Balance. Available online at: https://www.ons.gov.uk/peoplepopulationandcommunity/healthandsocialcare/conditionsanddiseases/articles/parentinginlockdowncoronavirusandtheeffectsonworklifebalance/latest (accessed October 10, 2020).

[B37] Perrewé P. Halbesleben J. Rosen C. (2012). The Role of the Economic Crisis on Occupational Stress and Well Being. Bingley: Emerald.

[B38] Robinson S. L. Rousseau D. M. (1994). Violating the psychological contract: a psychological contract perspective. J. Organ. Behav. 16, 245–298. 10.1002/job.4030150306

[B39] Safraz M. Ozturk I. Shah S. G. (2020). Coronavirus disease (COVID-19): the impact on psychology of sustainability, sustainable development, and global economy. Front. Psychol. 35185731 10.3389/fpsyg.2022.811863 PMC8854211

[B40] Saldaña J. (2021). The Coding Manual for Qualitative Researchers, 4th Edn. London: Sage.

[B41] Shack A. R. Meiyappan S. Grossman L. D. (2018). Improved self-esteem in artists after participating in the “building confidence and self-esteem toolbox workshop.” Front. Psychol. 9:1169. 10.3389/fpsyg.2018.01169 30026720 PMC6042157

[B42] Shehzad K. Xiaoxing L. Arif M. Rehman K. U. Ilyas M. (2020). Investigating the psychology of financial markets during covid-19 era: a case study of the us and european markets. Front. Psychol. 11:1924. 10.3389/fpsyg.2020.01924 33013504 PMC7494908

[B43] Shoss M. K. Horan K. A. DiStaso M. LeNoble C. A. Naranjo A. (2021). The conflicting impact of COVID-19's health and economic crises on helping. Group Organ. Manage. 46, 31–37. 10.1177/1059601120968704

[B44] Sonnentag S. (2001). Work, recovery activities, and individual well-being: a diary study. J. Occup. Health Psychol. 6, 196–210. 10.1037/1076-8998.6.3.196 11482632

[B45] Sonnentag S. (2005). Burnout research: adding an off-work and day-level perspective. Work Stress 19, 271–275. 10.1080/02678370500386473

[B46] Stoker J. I. Garretsen H. Soudis D. (2019). Tightening the leash after a threat: a multilevel event study on leadership behavior following the financial crisis. Leadersh. Q. 30, 199–214. 10.1016/j.leaqua.2018.08.004

[B47] Szulc J. M. (2020). Beyond quid pro quo: good soldiers and characteristics of their helping behaviours. Pers. Rev. 50, 560–574. 10.1108/PR-03-2019-0140

[B48] Tate L. E. (2020). Using rapid ethnography to unpack performances of community authenticity: an art festival case from Victoria, British Columbia. J. Plan. Educ. Res. 1–13. 10.1177/0739456X20920922. [Epub ahead of print].

[B49] Teng Y. M. Wu K. S. Lin K. L. (2020). Life or livelihood? mental health concerns for quarantine hotel workers during the COVID-19 pandemic. Front. Psychol. 11:2168. 10.3389/fpsyg.2020.02168 33041885 PMC7523406

[B50] Topping A. (2020). UK working mothers are 'sacrificial lambs' in coronavirus childcare crisis. The Guardian. UK Edition. Available online at: https://www.theguardian.com/money/2020/jul/24/uk-working-mothers-are-sacrifical-lambs-in-coronavirus-childcare-crisis

[B51] Tselikis P. (2001). Buoying morale and productivity during layoffs. Bus. Health 19, 23–27. 11771053

